# Pandora's Box

**DOI:** 10.1192/bji.2024.26

**Published:** 2024-11

## Big Brother watching?

Gone may be the days when referees’ and journal editors’ approval of papers for publication was enough. Post-publication scrutiny is on the way. Julian Nowogrodzki, a science writer and editor from Boston, Massachusetts, reports in *Nature* on a new, psychologist-driven movement that proposes a strategy to minimise errors in publications. The idea was initiated by Elson, a psychologist at the University of Bern, following the incidental finding of a number of errors in a highly influential paper, casting some doubt on its findings. The paper, published in 2010 by respected researchers from Harvard University in Cambridge, Massachusetts, on the subject of economics, ‘promoted austerity measures to reduce national debt’, and had a major influence on financial policies in Europe. The errors were only identified 3 years later, when a PhD student attempted to replicate the publication's findings. The authors of the paper cooperated in providing their data and admitted to the errors, although they maintained that their conclusions were sound.

This triggered the idea of Elson and colleagues to propose what they call the ERROR project, which will pay reviewers to check highly cited psychology papers, searching for errors in code, statistics and references cited. Reviewers will be paid for each paper reviewed and will in addition get a bonus for any errors they find, with the bonus payment being higher for bigger errors. This approach was modelled on the ‘bug bounty’ programmes that Microsoft, Google and other technology companies offer to hackers they employ to find and report possible weaknesses in their programs. Payments are also offered to the authors of publications for answering reviewers’ questions and for making their data available; extra payments are offered to them if no errors are found, or if any errors are only minor.

So far, only a few authors have agreed to have their studies reviewed. Finding reviewers is not straightforward, either. Despite the offer of reasonably attractive remuneration, many potential reviewers are likely to be in the early-career stage and may be wary of getting involved, owing to concerns about repercussions from senior academics in their field. However, the ERROR project has received funding and is gathering momentum, and it is hoped that it will be expanded to other disciplines in addition to psychology in the near future. Courses are being developed to teach error detection at a Master's level at psychology department of the University of Bern, and also at the Institute of Psychiatry, Psychology and Neuroscience at King's College, London. Elson and his colleagues hope that their project will flourish if funding bodies can be persuaded to pay for error reviews of publications they fund, arguing that this will be in everybody's interest, including that of the funders. Let's wait and see. In the meantime: researchers, do double-check your work; Big Brother may be watching.

Nowogrodzki J. Cash for catching scientific errors. Nature 2024; 632(8026): 942–3.39160228 10.1038/d41586-024-02681-2

## Gender equality: still an elusive dream?

In many research centres around the world, the number of women is increasing, but is their work valued as much as that of men? Unfortunately, it appears that by and large it is not. Existing research shows that women publish less, are less often the first author, and have fewer citations and lower h-indices than men. They are also less likely to be acknowledged for innovations or receive awards, particularly more prestigious ones.

A recent study examined gender differences in research quality evaluation, using data-sets from 30 countries (including New Zealand, Australia, Canada, countries of the European Union and the UK) and involving thousands of researchers, assessing success in securing funding and holistic research quality scores. As much as possible, they took into account the effects of various characteristics such as age, research institution and publishing patterns. For research quality evaluations, they used the performance-based research fund data-set of Aotearoa, New Zealand, which contains holistic research portfolio evaluations of every researcher in New Zealand in the years 2000–2006, 2007–2012 and 2013–2018.

Their results showed that researchers, both men and women, in male-dominated disciplines had higher funding success rates and higher research-quality schools compared with researchers of both genders in female-dominated disciplines. Although the data did not directly explain the reasons for these differences, the authors explored possible explanations for these findings. They speculated that reviewers may be biased against women and suggest that despite limited evidence, unconscious biases may affect academic metrics and practices such as the h-index, citations, authorship and peer review, as well as employment and career progression. They considered the possibility that women did lower-quality research, but their findings failed to support this. They also considered the possibility that owing to cultural norms, researchers of both genders have lower research outputs in female-dominated disciplines, or that women are attracted to disciplines with lower publication norms. They further discussed the possible role of job choice, i.e. the possibility that women choose to work in less well-paid areas or in disciplines that the educational system undervalues, and that within a discipline, women may tend to choose more interdisciplinary and applied research topics. The authors concluded that their data neither supported nor rejected job choice as an explanation. As regards bias against female-dominated disciplines, they again claim correlations rather than causation.

Whatever the specific reasons, which are multifactorial (social norms, psychological, biological, other), the fact remains that gender equality has a long way to go.

James A, Buelow F, Gibson L, Brower A. Female-dominated disciplines have lower evaluated research quality and funding success rates, for men and women. eLife 2024; 13: RP97613.39235445 10.7554/eLife.97613PMC11377033

## Time to stop politicising health research

Research and researchers have been targeted in the interest of politics rather than undergoing purely scientific scrutiny, as reported at least in the USA. The Covid pandemic highlighted this matter further. The US National Institutes of Health (NIH) director, Monica Bertagnolli, appointed in 2023, has now formally acknowledged that ‘some researchers feel the agency has unfairly targeted Asian and Asian American scientists, particularly those of Chinese descent, during a 6-year probe into unreported foreign ties among NIH grantees’. She says: ‘I recognize that certain government actions to protect against concerning activities by the PRC [People's Republic of China] … have had the unintended consequences of creating a difficult climate for our valued Asian American, Asian immigrant and Asian research colleagues who may feel targeted and alienated’. She announced that the NIH is now working with universities and other academic organisations to take actions, including guidelines, ‘to repair our relationships’.

Although this was acknowledged as a significant step forward, some feel that it doesn't go far enough. The Asian American Scholar Forum worked closely with the NIH on this announcement, and its executive director commented: ‘when policies are written down and specified, that helps increase transparency and reduce issues of racial biases’. Other scientists, however, were not convinced by Bertagnolli's assertion that ‘NIH's approach to addressing foreign interference has been and continues to be applied in a nondiscriminatory manner … that does not discriminate with respect to national origin or identity’, and they express disappointment that ‘she did not apologize or acknowledge that … NIH's probe has needlessly destroyed careers and lives’. Some of the targeted institutions also disagreed with Bertagnolli's claim that the NIH enforcing its long-established policies was not the reason for this, stating that there was pressure on their administration to take action, even though evidence for deliberate wrongdoing was shaky, because the institution was worried about losing NIH funding.

Surveys suggest that scientists born in China felt singled out by US investigative efforts and were made to feel unwelcome. A further statement was made by the NIH to clarify the summary of Bertagnolli's statement and explain that their efforts cover all foreign interference, not just that related to China. Building bridges is going to be difficult, and re-establishing confidence and trust will take time. How do the EU and UK authorities fare on this?

Kaiser J. NIH director expresses support for Asian researchers 6 years into its ‘China initiative’. *ScienceInsider*, 16 Aug 2024.

## Is there anybody there?

Are comatose patients unresponsive to commands capable of cognitive tasks? It is claimed that in many patients, functional magnetic resonance imaging (fMRI) and electroencephalography (EEG) can detect such brain activity. This phenomenon, called cognitive motor dissociation, had not been examined systematically in a large number of patients until a recent prospective cohort study, published in the *New England Journal of Medicine*. The researchers, at six international centres, collected clinical behavioural and task-based fMRI and EEG data from 353 adult subjects (median age 37.9 years) with disorders of consciousness, both with and without observable response to verbal commands. They assessed the presence or absence of observable responses to commands using the Coma Recovery Scale Revised (CRS-R). The median time between brain injury and assessment using the CRS-R was 7.9 months, and in one in four subjects this was done within 28 days of the brain injury. Brain trauma was the cause of coma in 50% of the cases. Data from fMRI only or EEG only were available for 65% of the participants, and both fMRI and EEG data were available for only 35%.

Cognitive motor dissociation was present in 25% of the subjects without an observable response to commands, and this was associated with younger age, longer time since injury and brain trauma as the aetiologic factor. Responses on task-based fMRI or EEG were present in 38% of the participants with an observable response to commands.

The authors concluded that one in four people in coma without an observable response to commands were able to perform a cognitive task on fMRI or EEG, compared with one in three of those with observable response to commands. The difference was not as great as one would expect, which may have implications in terms of care offered to non-responsive comatose patients and, very importantly, how long they are kept on life support. Clearly, more work is needed in this area.

Bodien YG, Allanson J, Cardone P, Bonhomme A, Carmona J, Chatelle C, et al Cognitive motor dissociation in disorders of consciousness. N Engl J Med 2024; 391: 598–608.39141852 10.1056/NEJMoa2400645PMC7617195

